# A Triangular Similarity Measure for Case Retrieval in CBR and Its Application to an Agricultural Decision Support System

**DOI:** 10.3390/s19214605

**Published:** 2019-10-23

**Authors:** Zhaoyu Zhai, José-Fernán Martínez Ortega, Pedro Castillejo, Victoria Beltran

**Affiliations:** Departamento de Ingeniería Telemática y Electrónica (DTE), Escuela Técnica Superior de Ingeniería y Sistemas de Telecomunicación (ETSIST), Universidad Politécnica de Madrid (UPM), C/Nikola Tesla, s/n, 28031 Madrid, Spain; jf.martinez@upm.es (J.-F.M.O.); pedro.castillejo@upm.es (P.C.); mv.beltran@upm.es (V.B.)

**Keywords:** triangular similarity measure, case retrieval, case-based reasoning, decision support system, sustainable agriculture

## Abstract

Case-based reasoning has been a widely-used approach to assist humans in making decisions through four steps: retrieve, reuse, revise, and retain. Among these steps, case retrieval plays a significant role because the rest of processes cannot proceed without successfully identifying the most similar past case beforehand. Some popular methods such as angle-based and distance-based similarity measures have been well explored for case retrieval. However, these methods may match inaccurate cases under certain extreme circumstances. Thus, a triangular similarity measure is proposed to identify commonalities between cases, overcoming the drawbacks of angle-based and distance-based measures. For verifying the effectiveness and performance of the proposed measure, case-based reasoning was applied to an agricultural decision support system for pest management and 300 new cases were used for testing purposes. Once a new pest problem is reported, its attributes are compared with historical data by the proposed triangular similarity measure. Farmers can obtain quick decision support on managing pest problems by learning from the retrieved solution of the most similar past case. The experimental result shows that the proposed measure can retrieve the most similar case with an average accuracy of 91.99% and it outperforms the other measures in the aspects of accuracy and robustness.

## 1. Introduction

Case-based reasoning (CBR) is defined as the process of solving new problems by matching and adapting cases that have been successfully managed before. The CBR approach mimics how humans would perform reasoning and learning. Thus, it seems to be a more psychologically plausible model of human reasoning [1]. This unique characteristic makes CBR a promising approach for building intelligent systems [2]. Due to its effectiveness and powerful reasoning capability, CBR has been applied to various fields such as healthcare [3,4,5], fault diagnosis [6,7,8], emergency response [9,10,11], and agricultural management [12,13,14].

**Figure 1:**
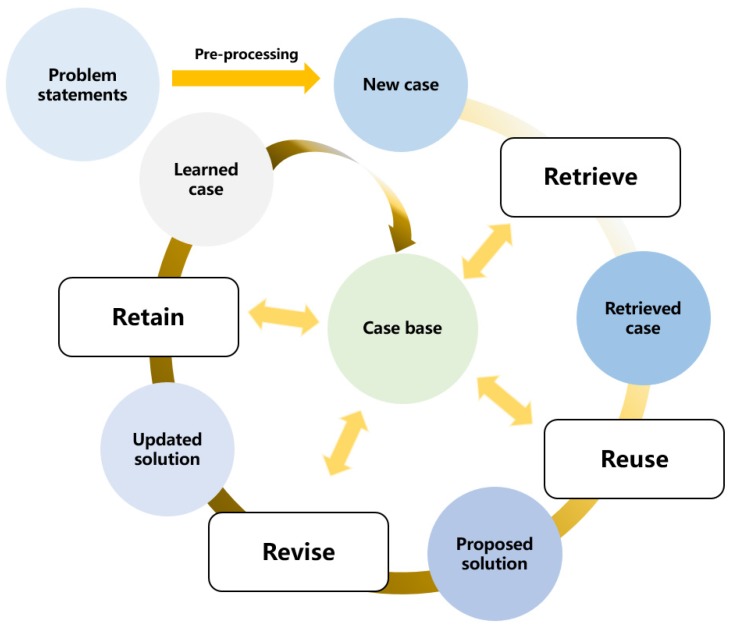
A generic workflow of case-based reasoning.

The case-based reasoning approach can be formalized as four main steps: retrieve, reuse, revise, and retain [15]. A generic workflow of CBR is presented in Figure 1.

In Figure 1, the problem statements are transformed into a new case after data pre-processing. The retrieval process compares the new case with historical ones for the purpose of identifying the most similar past case. The solution of this retrieved case is reused to solve the new case. In order to perfectly fit the new case, a revision process is required to update the proposed solution. After applying the updated solution, the solved case will be stored in the case base for further comparisons. Among these four steps, it is worth noting that the case retrieval process plays a key role because the rest of processes cannot proceed without successfully matching the most similar past case.

For case retrieval, many similarity measuring methods [16] have been used to evaluate the commonalities between cases, including angle-based and distance-based measures [17,18]. On the one hand, as one of the representative angle-based measures, cosine similarity measure [19] compares two non-zero vectors and calculates the cosine angle between them. It judges the similarity through their orientation, not magnitudes. A smaller cosine angle indicates that compared vectors are more similar. On the other hand, researchers have made tremendous contributions to distance-based similarity measures, especially the Euclidean distance [20], the Manhattan distance [21], the Chebyshev distance [22], and the Hamming distance [23]. Generally, the smaller the distance is, the more commonalities the compared vectors will have.

However, angle-based and distance-based measures may retrieve imprecise cases under certain extreme circumstances [24,25]. Thus, a novel triangular similarity measure (TSM) is presented in this paper to evaluate the commonalities between cases. The proposed measure not only considers the magnitude of vectors, but also their distances and magnitude differences for generating more accurate results. A case study on a CBR-based agricultural decision support system (ADSS) for pest management is explored to prove the effectiveness and robustness of the proposed measure. The triangular similarity measure is employed to retrieve similar past cases for providing farmers with quick decision support on agricultural activities.

The rest of this paper is organized as follows. In Section 2, related works on CBR-based ADSSs, angle-based and distance-based similarity measures are reviewed. According to detected drawbacks of current measures, the triangular similarity measure is explained in Section 3. A case study on applying the proposed measure to a CBR-based agricultural decision support system is explored in Section 4. Experimental results are presented in Section 5. Lastly, conclusions are drawn in Section 6.

## 2. Related Work

In this section, research works on applying the case-based reasoning approach to agricultural decision support systems are reviewed. It is demonstrated that how ADSSs could benefit from employing the CBR approach. Meanwhile, current states of angle-based and distance-based similarity measures are investigated for the purpose of detecting their drawbacks.

### 2.1. CBR Applications for Agricultural Decision Support Systems

An agricultural decision support system [26,27] can be defined as a human–computer system with the ability to utilize data from various resources such as climate, land use, and human labor. It aims at smoothening the decision-making processes and providing farmers with useful suggestions on agricultural activities, such as fertilization, irrigation, and pest management. With the help of sensing technologies and reasoning approaches, ADSSs are able to gather adequate data (e.g., sensor measurements, meteorological information, infrared images) and generate evidence-based knowledge.

As one of the most powerful reasoning approaches, CBR has been adopted by researchers in the aspect of agriculture for decades. Padma et al. [28] presented an intelligent decision support system for managing pest problems in an apple orchard. A hybrid algorithm was developed to optimize pest and disease protection decision-making processes by integrating case-based reasoning and database technologies. The nearest neighbor algorithm was used to determine the similarity between attributes of cases. Experimental results show that the accuracy of decision-making processes achieved 90.20%, thus the proposed ADSS can provide significant decision support for farmers in decision-making towards eco-friendly pest management practices. Evans et al. [14] mentioned that CBR is able to capture both experiential and expert knowledge to provide farmers with suggestions for potential action on agricultural practices. The advantage of adopting CBR in ADSSs is that domain experts do not need to ask a software engineer to update the knowledge base because step of retention in CBR can maintain the knowledge base on its own. Le Ber et al. [29] introduced a prototype of CBR and applied it to forecast miscanthus allocations. For case retrieval, a distance-based measure is used to compare the common attributes between the source and target problems. Then, the process of transformational adaptation [30] is conducted, starting from the solution of the selected source case and modifying it with respect to the differences between source and target cases. Sharaf-Eldeen et al. [31] developed a new case-based reasoning approach and improved the accuracy of case retrieval by range adaptation rules. The similarity between cases is calculated by a distance formula. The dissimilarity between attributes are used to generate the antecedent part of transformational adaptation rules while the differences between solutions of the compared cases become the consequent part of rules. The proposed approach was verified by a plant classification example. Over one hundred cases were considered in the case base and experimental results showed that the developed prototype achieved great accuracy in plant classification.

From the above research works, we conclude that the case-based reasoning approach has great potential in agricultural decision support systems. It is worth noting that case retrieval plays a key role in CBR. However, the similarity evaluation in the step of case retrieval remains to be further improved.

### 2.2. Angel-based Measures for Case Retrieval

Cosine similarity calculates the cosine angle between two non-zero vectors. It is widely used in case retrieval for case-based reasoning. The similarity of images, documents, and numeric values can be measured by the cosine similarity because all these data can be represented by vectors. The simplicity and effectiveness of cosine similarity measure has been acknowledged by the research community [32]. The cosine similarity of two vectors can be formularized in Equation (1) [33].

(1) $$Sim\left(x_{i},x_{i}^{\prime }\right)=\cos\left(\theta \right)=\;\frac{\sum_{i=1}^{n}x_{i}*x_{i}^{\prime }}{\sqrt{\sum_{i=1}^{n}x_{i}^{2}}*\sqrt{\sum_{i=1}^{n}{x_{i}^{\prime }}^{2}}}$$

where  θ is the angle between vectors x and $x^{\prime }$. $x_{i}$ and $x_{i}^{\prime }$ represents the *i*th attribute in the vectors x and $x^{\prime }$, respectively. The similarity between these two vectors will increase as cos(θ) increases.

Hassanien et al. [34] presented an automatic CBR based system for assessing water quality according to microscopic images of fish gills and livers. The cosine-based measure was used to find out a small number of cases from the case base with the highest similarity to the query. Compared with other similarity measures such as the Euclidean distance, the Canberra distance, and the squared chord distance, the proposed system achieved water quality prediction accuracy of 97.9% when using the cosine-based measure. More applications of the Cosine similarity measure are found in literature [35,36].

### 2.3. Distanec-Based Measures for Case Retrieval

Distance-based measures include the Euclidean distance, the Manhattan distance, the Chebyshev distance, and the Hamming distance. These similarity measures have various applications, such as information retrieval, classification, clustering and so on.

The Euclidean distance, also known as the Euclidean metric, calculates the straight-line distance between two points in Euclidean space. It has been employed to measure the similarity of numeric, interval, fuzzy data, etc. The formula of Euclidean distance is defined in Equation (2) as follows.

(2) $$Dist\left(x,\;x^{\prime }\right)=Dist\left(x^{\prime },x\right)=\sqrt{\sum_{i=1}^{n}{\left(x_{i}-x_{i}^{\prime }\right)}^{2}}$$

where Dist() is the distance between compared two vectors, while $x_{i}$ and $x_{i}^{\prime }$ represent the *i*th attribute in the vector x and $x^{\prime }$, respectively. The similarity of these two vectors will increase as Dist() decreases. The Euclidean distance similarity measure is adopted in literature [37,38].

The Manhattan distance between two vectors in N-dimensional space is the sum of the lengths of the line segments between two points onto the coordinate axes. Similar to the Euclidean distance, the Manhattan distance is also applicable for measuring the similarity of numeric, interval, fuzzy data, etc. The formula of Manhattan distance is defined in Equation (3) as follows.

(3) $$Dist\left(x,x^{\prime }\right)=Dist\left(x^{\prime },x\right)=\sum_{i=1}^{n}\left|x_{i}-x_{i}^{\prime }\right|$$

where Dist() is the distance between compared two vectors, while $x_{i}$ and $x_{i}^{\prime }$ represent the *i*th attribute in the vectors x and $x^{\prime }$, respectively. The similarity of these two vectors will increase as Dist() decreases. The Manhattan distance similarity measure is employed in literature [39,40].

The Chebyshev distance is defined as the greatest difference between two vectors among any coordinate dimensions. Its application is found in measuring the similarity of images, fuzzy sets, interval data, and so on. The formula of Chebyshev distance is defined in Equation (4) as follows.

(4) $$Dist\left(x,x^{\prime }\right)=Dist\left(x^{\prime },x\right)=\max\left(\left|x_{i}-x_{i}^{\prime }\right|\right)$$

where Dist() is the distance between compared two vectors, while $x_{i}$ and $x_{i}^{\prime }$ represent the *i*th attribute in the vectors x and $x^{\prime }$, respectively. The similarity of these two vectors will increase as Dist() decreases. Rashid [41] and Mousa and Yusof [42] used the Chebyshev distance for similarity measurement.

The Hamming distance is usually used to measure the similarity of strings. The length of strings should be equal. Hamming distance counts the minimum number of dis-matches between two strings. The formula of Hamming distance is defined in Equation (5) as follows.

(5) $$Dist\left(x,x^{\prime }\right)=Dist\left(x^{\prime },x\right)=\sum_{i=1}^{n}\left(x_{i}⊕x_{i}^{\prime }\right)$$

where Dist() is the distance between compared two vectors, while $x_{i}$ and $x_{i}^{\prime }$ represent the *i*th attribute in the vectors x and $x^{\prime }$, respectively. The symbol ⊕ represents the XOR operator. The similarity of these two vectors will increase as Dist() decreases. Applications of the Hamming distance similarity measure is detected in literature [43,44].

### 2.4. Drawbacks of Angle-Based and Distance-Based Measures

**Figure 2:**
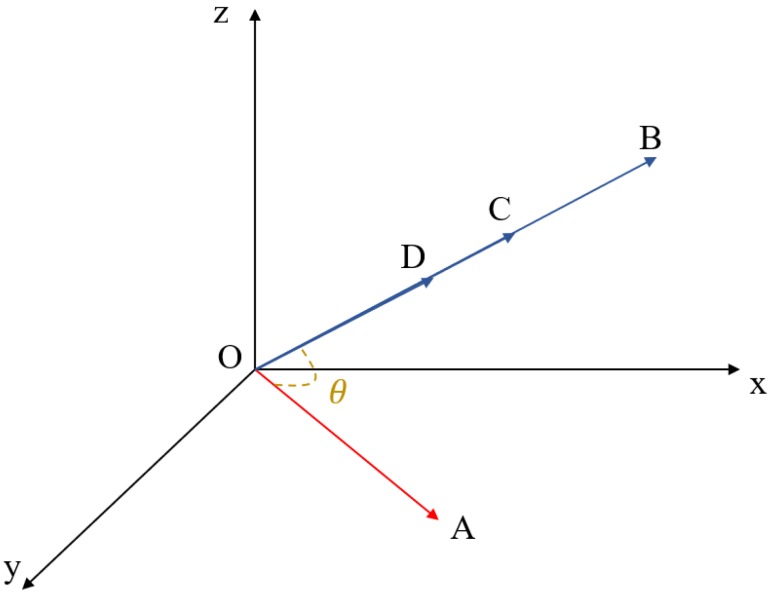
An extreme situation when measuring similarity with cosine similarity.

Though angle-based and distance-based measures are efficient and effective, their drawbacks cannot be ignored [45,46,47]. These measures may generate inaccurate results under certain extreme circumstances. For example, cosine similarity does not take the magnitudes of vectors into consideration, thus it may have difficulties when meeting the following situation shown in Figure 2.

In Figure 2, the target vector is $\vec{OA}$ and it is compared with vectors $\vec{OB}$, $\vec{OC}$, and $\vec{OD}$. On the one hand, though the angle between them is identical, there exists huge differences between their magnitudes. However, due to the same cosine similarity, vector $\vec{OA}$ is considered similar to vectors $\vec{OB}$, $\vec{OC}$, and $\vec{OD}$. On the other hand, cosine similarity between $\vec{OB}$, $\vec{OC}$, and $\vec{OD}$ all equals to one, meaning that these vectors are completely similar with each other. But the facts tell a different story. It is obvious that these vectors are not the same due to their magnitude differences. Thus, it is concluded that cosine similarity may match inaccurate cases in the step of case retrieval in CBR.

**Figure 3:**
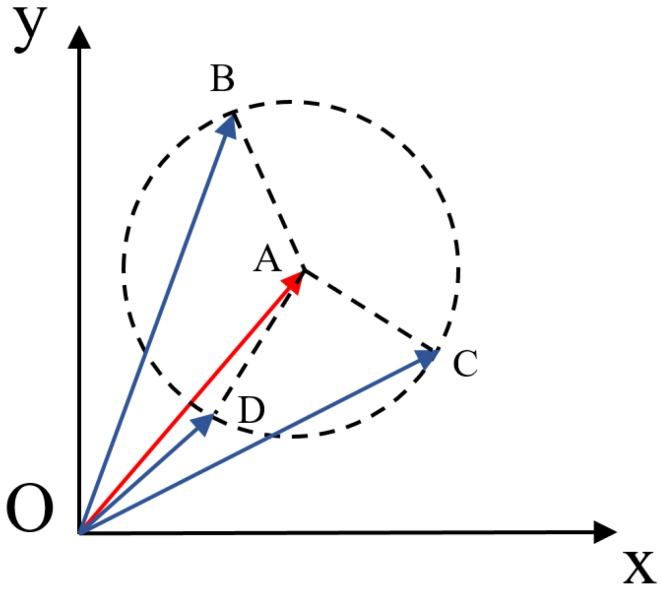
An extreme situation when measuring similarity with Euclidean distance.

In terms of distance-based measures, the Euclidean distance also has certain drawbacks [48,49,50]. It may have difficulties when the distances between several vectors are the same. This situation is shown in Figure 3.

In Figure 3, the target vector is $\vec{OA}$ and it is compared with vectors $\vec{OB}$, $\vec{OC}$, and $\vec{OD}$. Though all these vectors have different magnitudes and orientations, the distance between them is identical, indicating that vectors $\vec{OB}$, $\vec{OC}$, and $\vec{OD}$ are all similar to vector $\vec{OA}$. Meanwhile, it is acknowledged that each attribute in an individual vector may offer different contributions. However, the Euclidean distance treats all involved attributes equally. Thus, we can conclude that the Euclidean distance can be further improved to retrieve more accurate cases in CBR.

For the Manhattan distance and the Chebyshev distance, they both have the same drawbacks as the Euclidean distance [51,52,53,54,55,56]. When the distances between vectors are the same, these measures can hardly tell which vector is more similar to the target.

In conclusion, current similarity measures may be insufficient in the step of case retrieval for case-based reasoning. It is urgent to develop new similarity measures to improve retrieval accuracy and efficiency. Thus, a triangular similarity measure is proposed in this paper and explained in the next section.

## 3. Triangular Similarity Measure

After having a look in Figure 2 and Figure 3, a triangular similarity measure is proposed to overcome the drawbacks of angle-based and distance-based measures. The proposed measure takes the angle between two vectors and their magnitudes. When evaluating the similarity, a triangle is formed by two compared vectors. The area of this triangle is considered as a similarity metric. Meanwhile, for enhancing the robustness of TSM, a coefficient is designed in the formula. It considers the magnitude differences between two compared vectors. The smaller the triangular area is, the more similar the two vectors will be. The formula of TSM is explained in the next sub-sections in detail.

### 3.1. Formular of Triangular Similarity Measure

**Figure 4:**
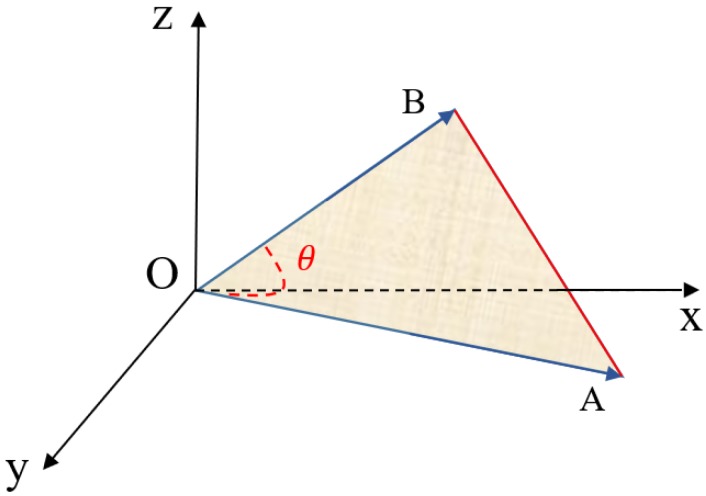
Two vectors $\vec{OA}$ and $\vec{OB}$ in three-dimensional space.

For two vectors in three-dimensional space, $\vec{OA}=\left(a_{1},a_{2},a_{3}\right)$ and $\vec{OB}=\left(b_{1},b_{2},b_{3}\right)$, they can form a triangle as follows in Figure 4.

In Figure 4, a triangle △AOB is formed by two vectors $\vec{OA}$ and $\vec{OB}$. The area of this triangle can be calculated by two sides and the included angle (SAS formula). Firstly, the magnitudes of the two vectors are calculated as follows.

(6) $$\left|\vec{OA}\right|=\sqrt{a_{1}^{2}+a_{2}^{2}+a_{3}^{2}}$$

(7) $$\left|\vec{OB}\right|=\sqrt{b_{1}^{2}+b_{2}^{2}+b_{3}^{2}}$$

The cosine value of these two vectors is calculated by Equation (1). For obtaining the included angle, the trigonometric inverse function $\cos^{-1}\theta$ can be employed. Then, the area of this triangle is calculated by the SAS formula as follows.

(8) $$S_{△\mathrm{AOB}}=\frac{\left|\vec{OA}\right|*\left|\vec{OB}\right|*\sin\left(\theta \right)}{2}$$

By employing TSM, we can obtain that the area of the triangle △AOD is much smaller than the triangle △AOC in Figure 3. Under this circumstance, it is concluded that vectors $\vec{OA}$ and $\vec{OD}$ have more commonalities than vectors $\vec{OA}$ and $\vec{OC}$, which is the expected result from common sense. Compared with distance-based measures, TSM is able to identify the most similar vector if when the distance between several vectors is the same. Thus, the proposed triangular similarity measure overcomes the drawbacks of distance-based measures.

### 3.2. Coefficient Design in Triangular Similarity Measure

**Figure 5:**
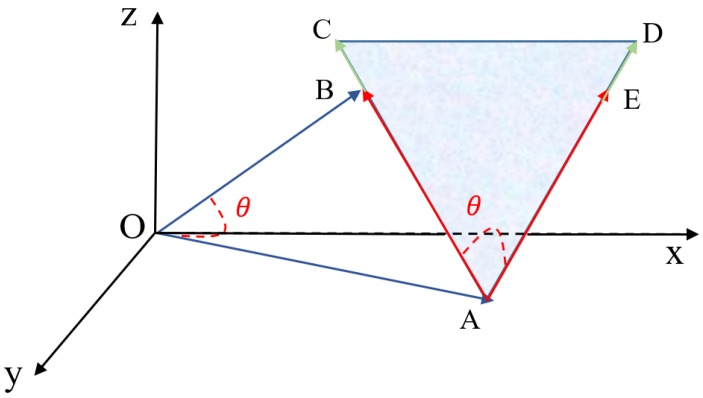
The coefficient triangle for vectors $\vec{OA}$ and $\vec{OB}$.

The robustness of TSM can be further enhanced for overcoming the drawbacks of angle-based similarity measures. In order to deal with the situation shown in Figure 2, a coefficient is designed to improve the similarity precision. We call it $K_{mag}$ because this coefficient mainly considers the magnitude difference between compared vectors. This coefficient is also formed by a triangle and its value is decided by calculating the area of the triangle. The coefficient for vectors $\vec{OA}$ and $\vec{OB}$ is shown in Figure 5.

As shown in Figure 5, the triangle △CAD is formed and its area represents the value of the coefficient for vectors $\vec{OA}$ and $\vec{OB}$. The angle (∠CAD) between $\vec{AC}$ and $\vec{AD}$ is the same as the angle ∠AOB. The magnitude of $\left|\vec{AB}\right|$ and $\left|\vec{AE}\right|$ equals to the Euclidean distance between vectors $\vec{OA}$ and $\vec{OB}$, while the magnitude of $\left|\vec{BC}\right|$ and $\left|\vec{ED}\right|$ equals to the magnitude difference between vectors $\vec{OA}$ and $\vec{OB}$. The magnitude of $\left|\vec{AC}\right|$ and $\left|\vec{AD}\right|$ is the sum of the Euclidean distance and the magnitude difference. The formula of $\left|\vec{AC}\right|$ and $\left|\vec{AD}\right|$ can be obtained by the following equation.

(9) $$\left|\vec{AC}\right|=\left|\vec{AD}\right|=\sqrt{{\left(a_{1}-b_{1}\right)}^{2}+{\left(a_{2}-b_{2}\right)}^{2}+{\left(a_{3}-b_{3}\right)}^{2}}+\left|\left|\vec{OA}\right|-\left|\vec{OB}\right|\right|$$

The first part of Equation (9) is the Euclidean distance between vectors $\vec{OA}$ and $\vec{OB}$, while the remaining part represents the magnitude difference between these two vectors. We detect that the Euclidean distance and the magnitude difference have a close relationship. When the magnitude difference decreases, so does the Euclidean distance. Thus, both factors are taken into consideration when designing the coefficient. The area of the triangle △CAD is also calculated by the SAS formula.

(10) $$K_{mag}=S_{△\mathrm{CAD}}=\frac{\left|\vec{AC}\right|*\left|\vec{AD}\right|*\sin\left(\theta \right)}{2}$$

With obtained areas of triangles △AOB and △CAD, the similarity between vectors $\vec{OA}$ and $\vec{OB}$ can be measured by the following equation.

(11) $$\mathrm{Sim}\left(\vec{OA},\vec{OB}\right)=K_{mag}*S_{△\mathrm{AOB}}$$

After multiplying the designed coefficient, TSM is complete and it is able to overcome the drawbacks of both angle-based and distance-based similarity measures.

For two vectors in N-dimensional space, $\vec{OA}=\left(a_{1},a_{2},\ldots ,a_{n}\right)$ and $\vec{OB}=\left(b_{1},b_{2},\ldots ,b_{n}\right)$, their similarity can be measured by the TSM formula defined as follows.

(12) $$Sim\left(\vec{OA},\vec{OB}\right)=\frac{{\left(\sqrt{\sum_{i=1}^{n}{\left(a_{i}-b_{i}\right)}^{2}}+\left|\sqrt{\sum_{i=1}^{n}a_{i}^{2}}-\sqrt{\sum_{i=1}^{n}b_{i}^{2}}\right|\right)}^{2}*\left[sin^{2}\left(\theta \right)+0.001\right]*\sqrt{\sum_{i=1}^{n}a_{i}^{2}}*\sqrt{\sum_{i=1}^{n}b_{i}^{2}}}{4}$$

In Equation (12), the sin(θ) is added by 0.001 for the following reason. When the compared two vectors are overlapped with each other, $Sim\left(\vec{OA},\vec{OB}\right)$ will equal to zero. As a consequence, the final result will be zero without this adjustment.

However, it may be difficult for human to understand the similarity by directly reading the triangle areas. It is better to adopt the inverse exponential function [57] to convert the area values into percentile values, ranging from 0 to 100%. Because the value presented by percentage can fit human notions better. This function is defined as follows.

(13) $$Sim{\left(\vec{OA},\vec{OB}\right)}_{\left[0,100\%\right]}=exp\left(-Sim\left(\vec{OA},\vec{OB}\right)\right)$$

A large value of $Sim{\left(\vec{OA},\vec{OB}\right)}_{\left[0,100\%\right]}$ means that vectors $\vec{OA}$ and $\vec{OB}$ are more similar. Only if vectors $\vec{OA}$ and $\vec{OB}$ are absolutely identical, the value of $Sim{\left(\vec{OA},\vec{OB}\right)}_{\left[0,100\%\right]}$ equals to one hundred percent. Because the value of $Sim\left(\vec{OA},\vec{OB}\right)$ equals to zero, indicating that the Euclidean distance between vectors and their magnitude differences are both zero.

## 4. Case Study: A CBR-Based Agricultural Decision Support System

In the previous section, the triangular similarity measure is explained in detail and it can be used for case retrieval in a CBR-based system. For verifying the effectiveness of the proposed TSM, we conduct a case study on applying the case-based reasoning approach to an agricultural decision support system. This ADSS aims at assisting farmers in managing pest problems under an eco-friendly manner. With the reduced chemical usages, soil fields can be greatly preserved, while applied chemicals can be precisely treated to infected and diseased crops.

The case study covers the following three parts: (1) problem statements for pest managements, (2) ADSS architecture, and (3) the process of retrieving similar past cases by the proposed TSM.

### 4.1. Problem Statements for Pest Management

The occurrence of pests has a close relationship with several factors, such as crop status, soil conditions, and meteorological information [58]. When managing pest problems, it is necessary to take pest, crop, and environment data into account. Thus, pest problems are defined by these three categories of attributes.

Firstly, different pest problems usually require specific chemical treatments. For example, rice growers may apply VIRTAKO or Prevathon for removing the eggs of stem borers [59]. Meanwhile, pest quantity has a great influence on applied dosages of chemicals. Therefore, it is necessary to obtain pest details beforehand.

Secondly, crop data are also essential. For example, the planting density of crops may affect the dilution concentration of chemicals [60]. Meanwhile, crops are sensitive to applied chemicals due to their growth stages. Toxic chemicals may be too strong, even lethal to those crops which are at the seed stage. Therefore, crops details are also important for the process of decision-making.

Lastly, environment variables play a significant role in pest management. The performance of applying chemical treatments may be easily affected by environmental changes. For example, the performance will decrease when it is raining. Moreover, it is not possible to use unmanned aerial vehicles (UAVs) for spraying the chemicals when the wind is too strong [61].

Conclusively, pest, crop, and environment variables are all considered in pest management in Table 1.

**Figure 6:**
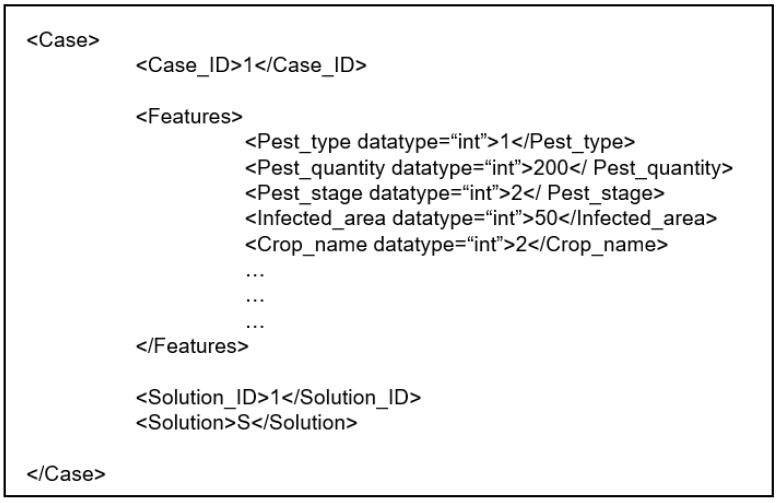
An example of the pair representation in an agricultural decision support system (ADSS).

Since the case-based reasoning approach is applied to the agricultural decision support system, problem statements of pest management should be represented by cases. In this paper, we adopt the feature-value (attribute-value) pair representation to define each case [63]. Meanwhile, each past case has its own solution. An example of the pair representation is shown in Figure 6 as follows.

### 4.2. Agricultural Decision Support System Overview

**Figure 7:**
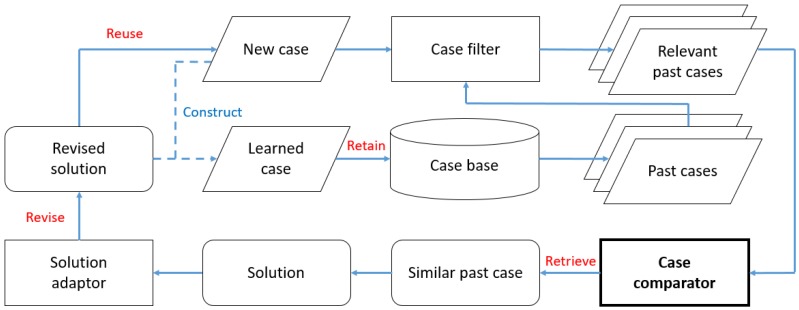
Framework of case-based reasoning (CBR)-based ADSS.

This agricultural decision support system aims at assisting farmers in managing pest problems. After a new pest problem is reported by farmers, the ADSS tries to identify the most similar past case from the case base and reuse the retrieved solution for resolving the new case. The adopted framework of ADSS [64] is shown in Figure 7.

In Figure 7, the ADSS consists of three main components: a case filter, a case comparator, and a solution adaptor. Firstly, the case filter is used to remove irrelevant past cases. As stated in Section 4.1, each past case contains information about pests and crops. The pest type and crop name are selected as identifiers. For example, a new case is in regards to managing a rice planthopper problem for rice. Under this circumstance, the pest type is “rice planthopper” and the crop name is “rice”. The case filter component selects past cases from the case base which match these two identifiers. The process of case filtering enables the ADSS to improve the efficiency of similarity measurements because the number of past cases selected for comparisons is reduced. Secondly, the new case and relevant past cases are treated as input to the case comparator, which is the core of the ADSS, because steps of revision, reuse, and retention cannot be further processed without successful case retrieval. The case comparator component employs the proposed triangular similarity measure to match the most similar past case. Thirdly, the solution adaptor component is used to revise the retrieved solution for fitting the situation of the new case better. Lastly, learned cases are constructed by problem statements of the new case and the revised solution. These data are retained in the case base for further comparisons.

### 4.3. Case Retrieval in ADSS by TSM

The process of case retrieval in the CBR-based ADSS is performed by the case comparator component. After case filtering, relevant cases are obtained. Since feature-value pair representations are employed, each case can be considered as a vector in N-dimensional space. The new case is compared with past cases one by one, using Equation (12). After computing areas of all formed triangles, these area values are standardized and converted into percentile values by Equation (13). The past case with the highest percentile value is chosen as the most similar one and its solution is retrieved for further revision. It is worth mentioning that each attribute of cases is equally weighted [65], indicating that each attribute has the same priority.

## 5. Experiments and Discussion

After introducing the problem statements of pest management and the ADSS overview, the proposed triangular similarity measure is verified in this section. Firstly, the experiment settings are presented. In total, one thousand past cases were stored in the case base and 300 new cases were prepared based on three categories of attributes defined in Section 4.1 for testing purposes. Then, we analysed the experimental results to verify whether the proposed TSM could retrieve the most similar past case or not. Lastly, the accuracy of TSM was compared with typical Euclidean distance and cosine similarity measures for demonstrating its effectiveness and superiority.

### 5.1. Experiment Settings

As mentioned in the previous section, this case study emphasises on applying the case-based reasoning approach to an agricultural decision support system for pest management. Two pests are considered in the case study: the rice planthopper (RP) [66] and the Chilo suppressalis (CS) [67]. In terms of crops, we focus mainly on managing pest problems for rice. In total, one thousand past cases were stored in the case base, half for RP and half for CS. The case base can be found at the following link: https://github.com/ZhaoyuZHAI/Case-base/blob/master/past%20case. Meanwhile, 300 new cases were designed for test purposes, 150 for RP and 150 for CS. Some data of new cases are given in Table 2. Each new case is compared with past cases, respectively. For now, we play with simulated data. The pest, crop, and environment data are randomly generated within a reasonable interval. For example, the attribute of crop planting density is generated from 180–525 seeds/m^2^ [68]. Since TSM is developed within a European project, entitled Aggregate Farming in the Cloud (AFarCloud), we expect to receive data from real fields as soon as the sensor deployment is complete.

### 5.2. Experimental Results

The desired output from the case comparator component is the ID number of the most similar past case and its corresponding similarity compared with the new case. The experimental result is shown in Table 3.

As shown in Table 3, new cases are matched with the most similar past cases, respectively. The attribute visualization of compared new and retrieved past cases in Table 3 is given in Appendix A at the end of this manuscript. From the visualization, it is determined that the attributes of retrieved past cases have great commonalities with the new cases correspondingly. Thus, the retrieved cases are considered similar to the new cases. The retrieved solutions of past cases can be used to resolve the new cases.

**Figure 8:**
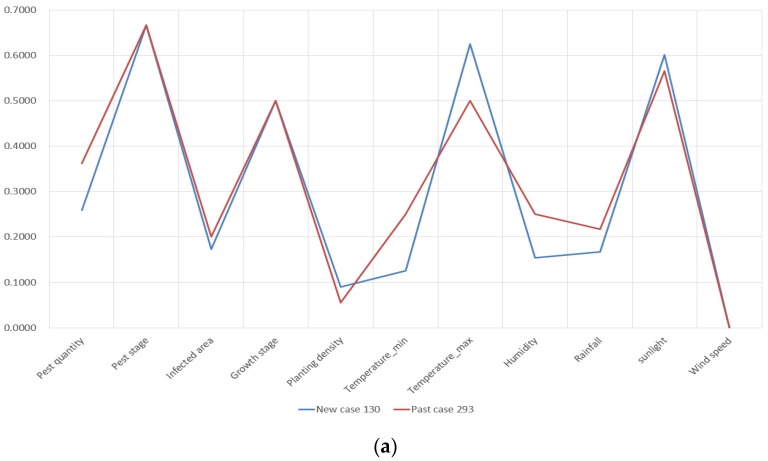

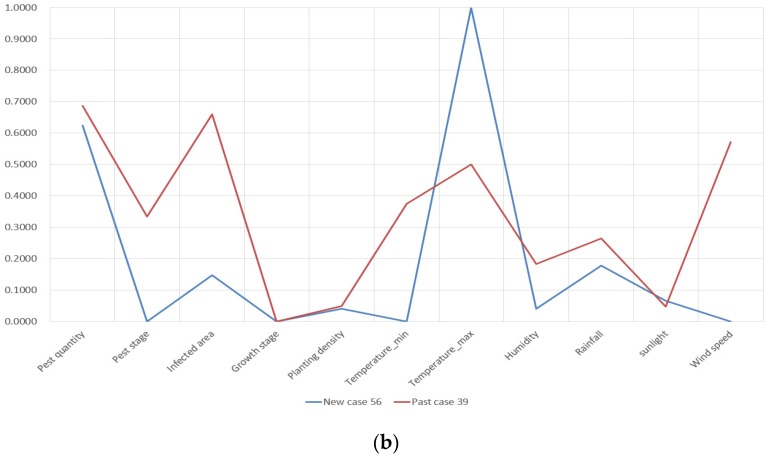
The similarity of retrieved cases: (**a**) the best situation; (**b**) the worst situation.

As 300 new cases are tested, 300 past cases are retrieved correspondingly. The mean similarity value of retrieved cases achieves 91.99%. The maximum and minimum similarity value of retrieved cases is 99.67% and 79.20%, respectively, as shown in Figure 8.

In Figure 8a, we can see that each attribute of New Case 130 and Past Case 293 matches each other with minor deviations. Thus, these two cases are considered highly similar. The retrieved solution of Past Case 293 has great potential in resolving the situation of New Case 130. In Figure 8b, the retrieved Past Case 39 does not match New Case 56 very well. The reason for this poor performance is that the case base contains only 1000 cases and it does not cover a situation like New Case 56. However, it is promising for the CBR-based ADSS to achieve an improvement because the case base will include more cases with time. As a consequence, future cases can have a greater chance for identifying similar ones in the case base and retrieving an effective solution.

By employing TSM, the most similar past case can be precisely retrieved. The pest, crop, and environment data of the retrieved past case have great commonalities with the new case. The successful case retrieval establishes a good foundation for the rest of processes in case-based reasoning. Meanwhile, the retrieved solutions of past cases are indeed useful guidance for farmers to manage pest problems. Farmers can eliminate pests by referring to solutions of past cases which have been successfully managed before.

The main focus of this paper lies on proposing a novel triangular similarity measure for case retrieval in case-based reasoning. Therefore, the rest of processes like revision is not considered in this paper.

### 5.3. Comparisons with Typical Similarity Measures

**Figure 9:**
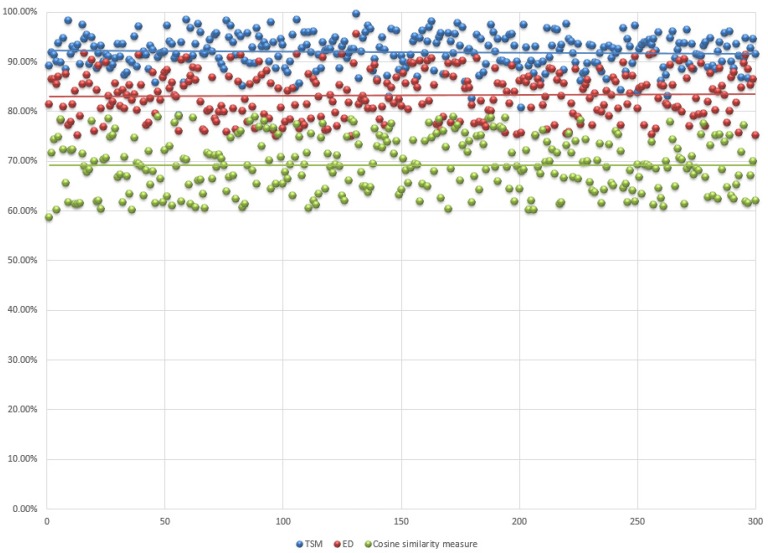
The result of all 300 retrieval tasks by TSM, ED, and cosine similarity measure.

In this section, the proposed triangular similarity measure is compared with two typical similarity measures: the Euclidean distance (ED) and cosine similarity. The experiment settings remain the same. However, instead of using Equation (12), the similarity between compared vectors is measured by Equation (1) and (2), respectively. Each attribute is also equally weighted. The inverse exponential function is still used in these two measures for presenting the final results. We select retrieval similarity as the main evaluation criteria for this comparison. The result of all 300 retrieval tasks is shown in Figure 9, while the comparative result of selected cases is shown in Table 4 as follows.

In Figure 9, blue, red, and green points represent the similarity value of past cases which are retrieved by TSM, ED, and cosine similarity measures, respectively. The average similarity value of these three measures is 91.99%, 83.20%, and 69.21%, respectively. From this point of view, we can conclude that TSM can retrieve the most similar case with a greater accuracy. For further demonstrating the accuracy of retrieved past case, a particular new case will be presented later which all three measures retrieved different past cases.

In Table 4, the result is presented by “Past case ID (similarity value)”. Under the column of TSM, it shows that TSM is able to retrieve the most similar past cases with the highest accuracy. In terms of the Euclidean distance measure, it fails to match the most similar cases for New Cases 151 and 154. With regards to the cosine similarity measure, it obviously fails in all cases. Conclusively, the retrieval similarity of TSM outperforms both typical similarity measures. TSM achieves the highest performance in case retrieval for all ten test cases. Though the Euclidean distance measure is able to achieve the same similar past cases as TSM, its similarity accuracy is worse than TSM. For the cosine similarity measure, it is not suitable for such case retrieval tasks. Moreover, in Section 3, we have proved that the robustness of TSM is better than the Euclidean distance and cosine similarity measures. TSM can always provide correct results even in extreme situations.

**Figure 10:**
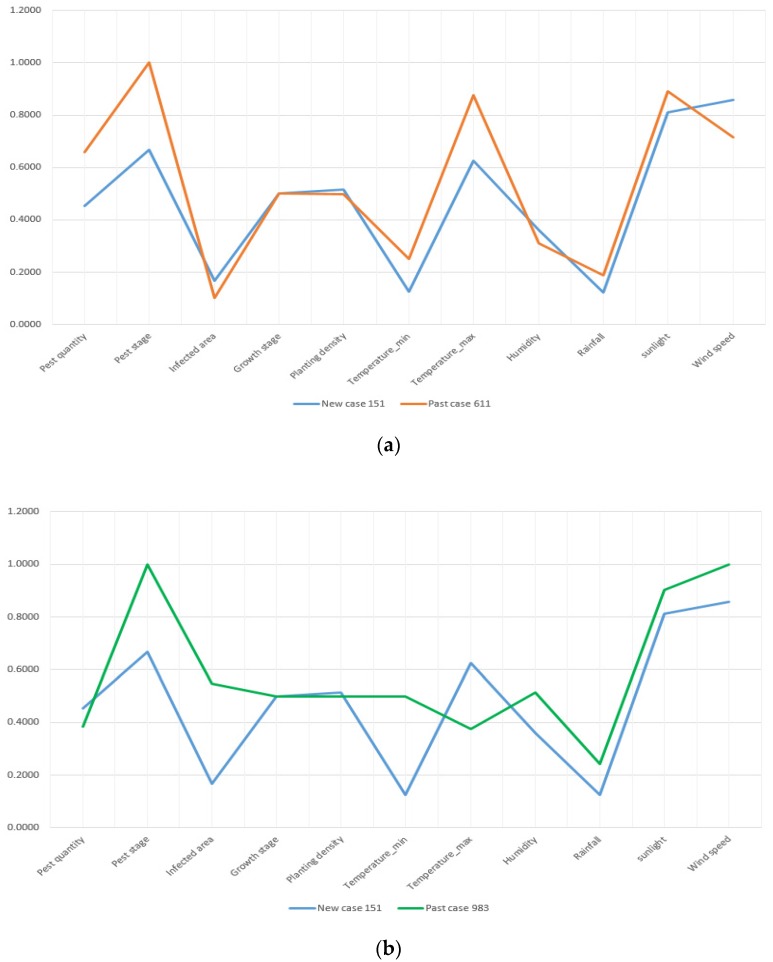

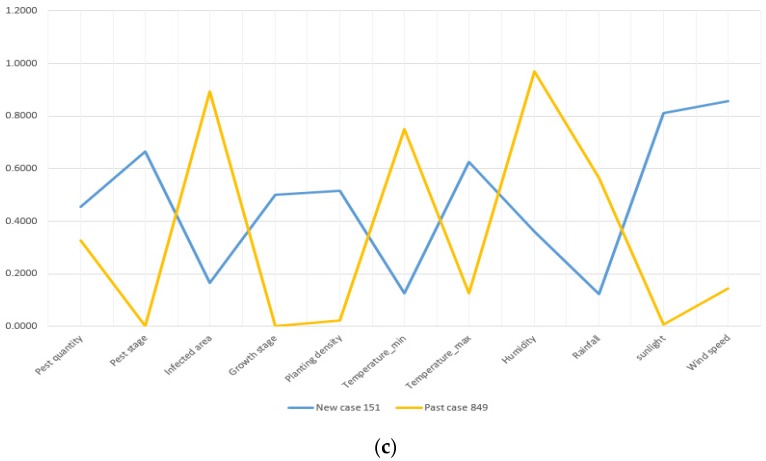
Comparative results of similarity accuracy: (**a**) past case retrieved by TSM; (**b**) case retrieved by ED; and (**c**) past case retrieved by cosine similarity measure.

For demonstrating the similarity accuracy of these measures, we select New Case 151 as an example. The standardized attributes of pest quantity, pest stage, infected area, growth stage of crops, planting density, temperature, humidity, rainfall, sunlight, and wind speed of the new case and retrieved past cases are presented by line charts in Figure 10.

In Figure 10, the past case retrieved by TSM is obviously more similar with the compared case than those retrieved by the ED and cosine similarity measures. Because the attribute deviation is smaller and the data trending between New Case 151 and Past Case 611 is more compliant. In terms of Past Case 983, its attributes of infected area, the minimum temperature, and humidity do not match New Case 151 perfectly. With regards to Past Case 849, retrieved by the cosine similarity measure, its attributes have a major difference from the target case.

Furthermore, we have compared the average precision of all these three similarity measures by Equation (14) [69].

(14) $$\mathrm{Average}\;\mathrm{precision}=\;\frac{TP}{TP+FP}\;\left(\%\right)$$

where *TP* means true positive and *FP* stands for false positive.

The average precision of all these three similarity measures are presented in Table 5 as follows. This criteria indicates the correctness of case retrieval.

In Table 5, the average precision of TSM achieves the highest value at 96.33%, while the ED similarity measure ranks second at 94.00%, following by the cosine similarity measure in third place (42.67%). Thus, the effectiveness and accuracy of TSM has been proved through comparative experiments.

## 6. Conclusions and Future Work

This paper focuses on proposing a triangular similarity measure for case retrieval in case-based reasoning systems. By reviewing current research works, we detected some drawbacks of angle-based and distance-based similarity measures. Typical measures may have difficulties in case retrieval under certain extreme situations. Though several cases sometimes have the same angle value or distance value, huge differences may exist among these cases, especially for their magnitude differences. Thus, a triangular similarity measure is presented to overcome these issues and provide users with a more accurate retrieval result. Meanwhile, a dynamic coefficient is designed to enhance the robustness of TSM. For verifying the proposed TSM, a case study on a CBR-based agricultural decision support system for pest management is conducted. TSM is adopted in the case comparator component for measuring the similarity between cases. The experimental result showed that TSM is able to retrieve the most similar past case with an average accuracy of 91.99%. By revising and reusing the retrieved solution, the ADSS can provide farmers with quick and accurate decision-making suggestions on managing pest problems for rice. Lastly, TSM is compared with typical Euclidean distance and cosine similarity measures. The effectiveness and accuracy of TSM is proved through the comparative experiments. Under the same experiment settings, the past case retrieved by TSM was more similar than those retrieved by the Euclidean distance and cosine similarity measures. By learning from an accurate past case, the rest of the processes, such as solution adaptation, may be easier and more efficient because data of retrieved past case have greater commonalities with the new case than those retrieved by typical similarity measures. Besides applying the proposed measure in CBR-based ADSS, it is also promising to employ this measure in any CBR-based systems like CBR-based clinical systems for disease diagnosis, CBR-based mechanical systems for fault detection, CBR-based emergency systems for emergency response, and so on.

Though the proposed triangular similarity measure has great potential in case retrieval, further developments should be continued for the purpose of improving its performance. Firstly, equal weights may not fully reflect the characteristics of cases. Naturally, weights address the relative importance of each attribute. Those important attributes should be assigned with larger weights during the process of decision-making. Secondly, for improving the efficiency of case retrieval, similar past cases in the case base may be associated with each other. Thus, the structured case representation is especially helpful to connect similar cases. Once a past case is compared with the new case, the similar ones will be preferentially extracted from the case base for comparisons. Thus, the number of visited cases can be reduced. Thirdly, there exists semantic relations within cases. Ontology and semantic reasoning techniques could be included in future work for improving the performance of case retrieval. Lastly, it is promising to verify the proposed TSM and CBR-based ADSS with collected data in real fields.

## Figures and Tables

**Table 1:** List of attributes considered in pest management [62].

Category	Attribute Name	Attribute Unit	Content
Pest	Pest type	/	Name of pests
	Pest quantity	Unit	Number of pests
	Pest stage	/	Life cycle of pests
	Infected area	m^2^	Area of infected districts
Crop	Crop name	/	Name of crops
	Growth stage	/	Life cycle of crops
	Planting density	Seeds/m^2^	Seeds density in an area
Environment	Temperature	Celsius degree	Minimum and maximum temperature
	Humidity	/	Amount of water vapor in air
	Rainfall	/	Possibility of rainfall
	Sunlight	Lux	Daylight during the daytime
	Wind speed	Degree	Wind flow velocity

**Table 2:** Some data of new cases for test purposes.

New Case ID.	1	2	3	4	5	151	152	153	154	155
Pest type	RP	RP	RP	RP	RP	CS	CS	CS	CS	CS
Pest quantity	335	320	384	390	323	309	356	334	458	345
Pest stage	Pupae	Adult	Larvae	Larvae	Pupae	Larvae	Adult	Egg	Larvae	Egg
Infected area	77	56	87	109	76	75	146	168	95	157
Crop name	Rice	Rice	Rice	Rice	Rice	Rice	Rice	Rice	Rice	Rice
Growth stage	Repro	Ripen	Vege	Ripen	Vege	Repro	Ripen	Repro	Repro	Repro
Planting density	473	272	479	367	345	358	285	274	217	355
Temperature	[21,30]	[16,30]	[15,29]	[21,32]	[16,32]	[16,29]	[20,25]	[18,32]	[22,32]	[17,24]
Humidity	57.49%	60.27%	87.06%	35.22%	25.73%	36.04%	65.68%	43.35%	31.02%	46.05%
Rainfall	72.15%	37.39%	17.34%	82.90%	51.26%	12.50%	61.93%	25.79%	80.42%	14.48%
Sunlight	4830	831	3338	3273	1888	4146	3972	2680	995	4742
Wind speed	2	4	3	1	2	7	1	8	3	5

**Table 3:** Some retrieved similar past cases and their corresponding similarity values.

New Case ID	1	2	3	4	5	151	152	153	154	155
Past case ID	148	412	378	387	38	611	700	918	580	672
Similarity	88.66%	91.82%	91.43%	89.92%	93.68%	89.97%	88.73%	89.72%	91.40%	94.01%

**Table 4:** Comparative result of three similarity measures in selected cases.

New Case ID	TSM	Euclidean Distance	Cosine Similarity
1	148 (**88.66%**)	148 (81.84%)	237 (58.74%)
2	412 (**91.82%**)	412 (86.48%)	89 (71.57%)
3	378 (**91.43%**)	378 (86.41%)	253 (74.34%)
4	387 (**89.92%**)	387 (85.43%)	106 (60.16%)
5	38 (**93.68%**)	38 (86.89%)	183 (74.76%)
151	611 (**89.97%**)	983 (82.74%)	849 (70.53%)
152	700 (**88.73%**)	700 (85.63%)	732 (68.09%)
153	918 (**89.72%**)	918 (84.44%)	755 (65.62%)
154	580 (**91.40%**)	568 (84.48%)	772 (68.65%)
155	672 (**94.01%**)	672 (85.38%)	421 (74.23%)

**Table 5:** The average precision of TSM, ED, and cosine similarity measures.

Similarity Measure	TSM	ED	Cosine Similarity Measure
Average precision	96.33%	94.00%	42.67%

**Figure A1:**
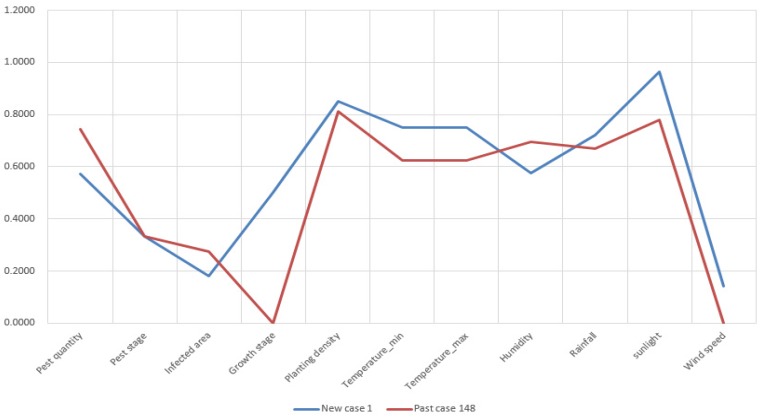
Attribute visualization of new case 1 and retrieved past case 148.

**Figure A2:**
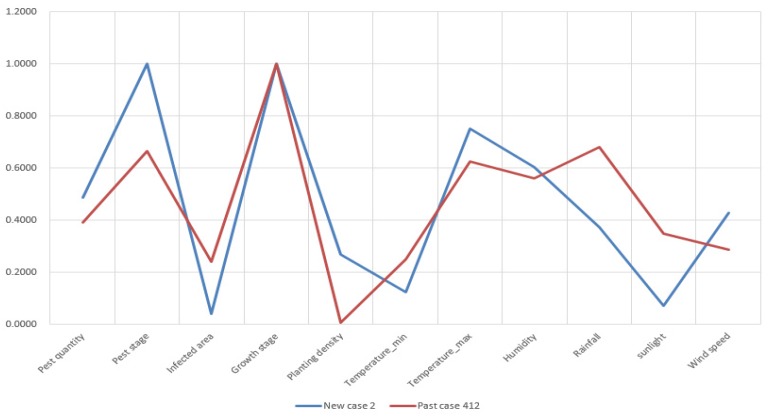
Attribute visualization of new case 2 and retrieved past case 412.

**Figure A3:**
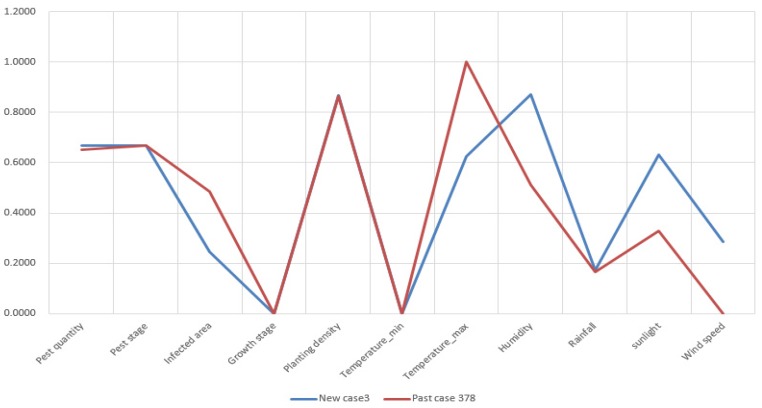
Attribute visualization of new case 3 and retrieved past case 378.

**Figure A4:**
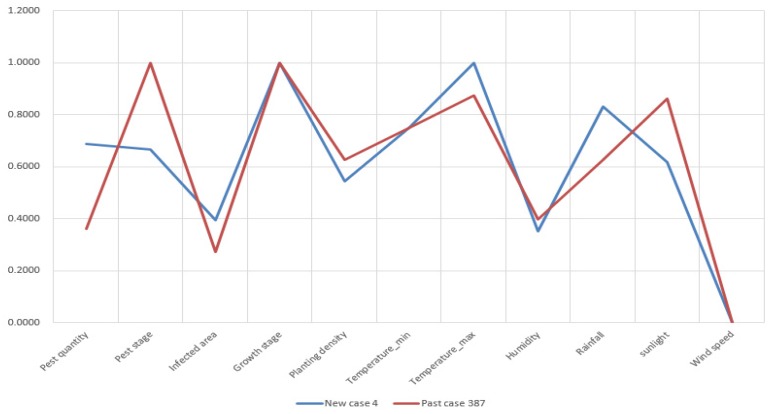
Attribute visualization of new case 4 and retrieved past case 387.

**Figure A5:**
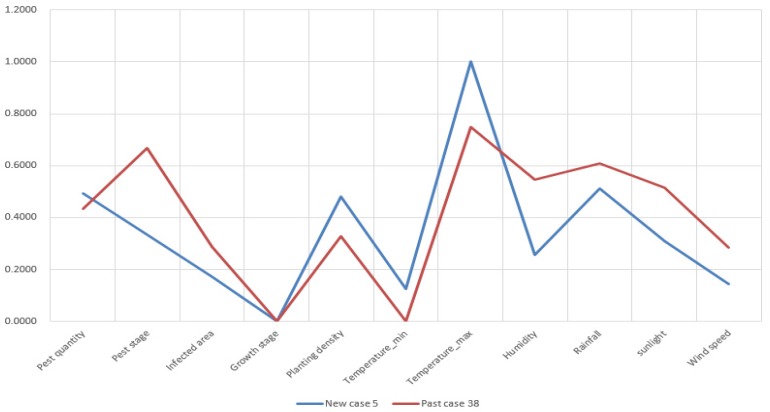
Attribute visualization of new case 5 and retrieved past case 38.

**Figure A6:**
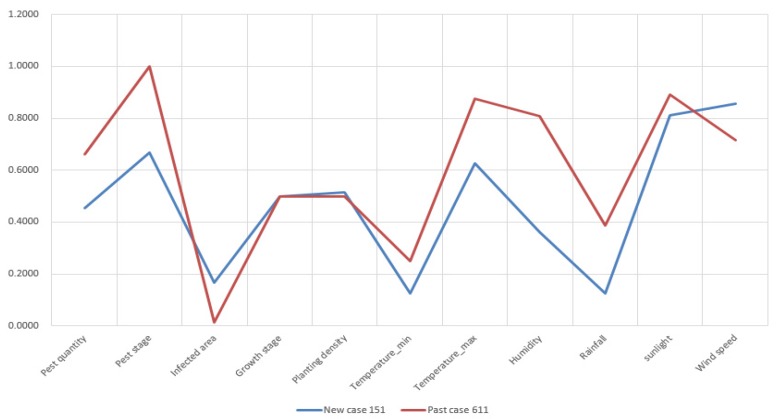
Attribute visualization of new case 151 and retrieved past case 611.

**Figure A7:**
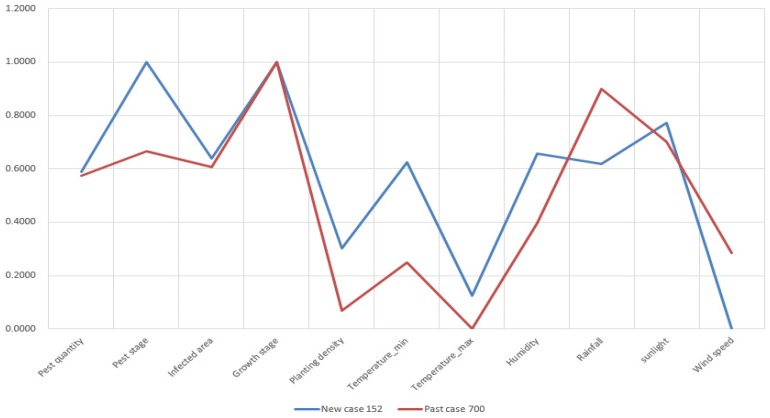
Attribute visualization of new case 152 and retrieved past case 700.

**Figure A8:**
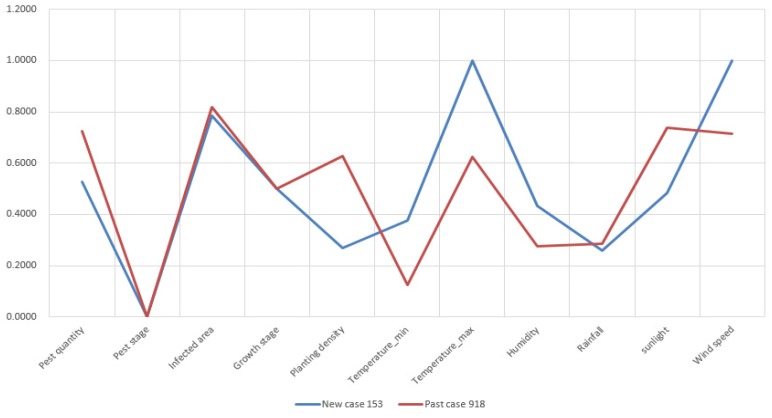
Attribute visualization of new case 153 and retrieved past case 918.

**Figure A9:**
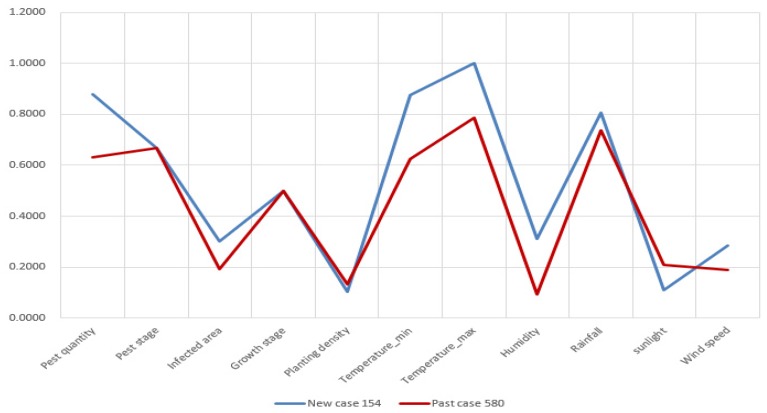
Attribute visualization of new case 154 and retrieved case 580.

**Figure A10:**
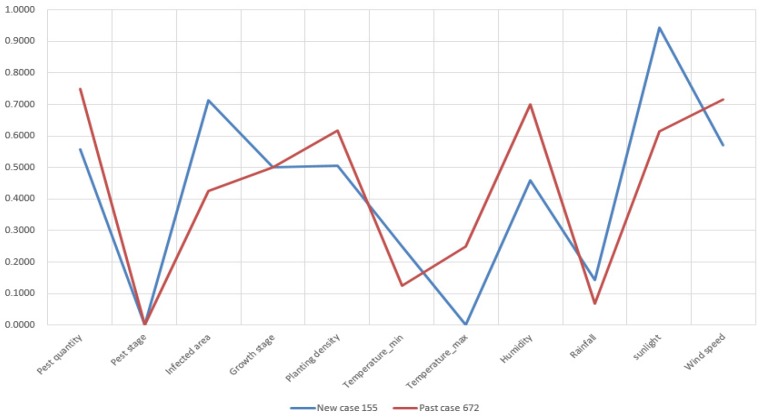
Attribute visualization of new case 155 and retrieved past case 672.

## Appendix A

This appendix contains the attribute visualization of compared cases in Table 3.
